# Comparing marginal structural models to standard methods for estimating treatment effects of antihypertensive combination therapy

**DOI:** 10.1186/1471-2288-12-119

**Published:** 2012-08-06

**Authors:** Tobias Gerhard, Joseph AC Delaney, Rhonda M Cooper-DeHoff, Jonathan Shuster, Babette A Brumback, Julie A Johnson, Carl J Pepine, Almut G Winterstein

**Affiliations:** 1 Institute for Health, Health Care Policy and Aging Research, Rutgers University, New Brunswick, NJ, USA; 2 Department of Pharmacy Practice and Administration, Ernest Mario School of Pharmacy, Rutgers University, Piscataway, NJ, USA; 3 Department of Pharmaceutical Outcomes and Policy, College of Pharmacy, University of Florida, Gainesville, FL, USA; 4 Department of Epidemiology, University of Florida, Gainesville, FL, USA; 5 Department of Biostatistics, University of Florida, Gainesville, FL, USA; 6 Department of Pharmacotherapy and Translational Research, College of Pharmacy, University of Florida, Gainesville, FL, USA; 7 Division of Cardiovascular Medicine, College of Medicine, University of Florida, Gainesville, FL, USA; 8 Department of Health Outcomes and Policy, College of Medicine, University of Florida, Gainesville, FL, USA

**Keywords:** Blood pressure, Hypertension, Time-dependent confounding, Marginal structural models

## Abstract

**Background:**

Due to time-dependent confounding by blood pressure and differential loss to follow-up, it is difficult to estimate the effectiveness of aggressive versus conventional antihypertensive combination therapies in non-randomized comparisons.

**Methods:**

We utilized data from 22,576 hypertensive coronary artery disease patients, prospectively enrolled in the INternational VErapamil-Trandolapril STudy (INVEST). Our post-hoc analyses did not consider the randomized treatment strategies, but instead defined exposure time-dependently as aggressive treatment (≥3 concomitantly used antihypertensive medications) versus conventional treatment (≤2 concomitantly used antihypertensive medications). Study outcome was defined as time to first serious cardiovascular event (non-fatal myocardial infarction, non-fatal stroke, or all-cause death). We compared hazard ratio (HR) estimates for aggressive vs. conventional treatment from a Marginal Structural Cox Model (MSCM) to estimates from a standard Cox model. Both models included exposure to antihypertensive treatment at each follow-up visit, demographics, and baseline cardiovascular risk factors, including blood pressure. The MSCM further adjusted for systolic blood pressure at each follow-up visit, through inverse probability of treatment weights.

**Results:**

2,269 (10.1%) patients experienced a cardiovascular event over a total follow-up of 60,939 person-years. The HR for aggressive treatment estimated by the standard Cox model was 0.96 (95% confidence interval 0.87-1.07). The equivalent MSCM, which was able to account for changes in systolic blood pressure during follow-up, estimated a HR of 0.81 (95% CI 0.71-0.92).

**Conclusions:**

Using a MSCM, aggressive treatment was associated with a lower risk for serious cardiovascular outcomes compared to conventional treatment. In contrast, a standard Cox model estimated similar risks for aggressive and conventional treatments.

**Trial registration:**

Clinicaltrials.gov Identifier: NCT00133692

## Background

Antihypertensive therapy commonly involves multi-drug treatment strategies that are initiated and continuously adapted based on clinical characteristics, blood pressure response, and adverse drug reactions. Due to the complexity of such treatment regimens with respect to drug combinations and doses, evidence from randomized controlled trials (RCTs) is often insufficient to inform common clinical decisions in antihypertensive therapy. There has been increasing interest in the question of whether combination antihypertensive therapies lead to improved clinical outcomes [1-4]. Furthermore, emerging evidence suggests the benefit of aggressive therapy may be limited to those in specific high risk groups, [5,6] which makes the focus on such populations, such as elderly patients with prevalent coronary artery disease (CAD), of particular interest.

Observational studies, including post hoc analyses of RCTs, are well suited to supplement the evidence base from RCTs, at least until a RCT is conducted to more carefully test the effect of aggressive therapy in clinically important sub-populations [1]. However, concerns exist that findings from such nonrandomized studies may be biased due to confounding by blood pressure and other clinical characteristics. For example, because more aggressive combination treatments are more likely to be initiated in patients with uncontrolled blood pressure, it is likely that the beneficial treatment effects of such combination therapies will be underestimated. This potential underestimation occurs because uncontrolled blood pressure simultaneously increases the likelihood of treatment with aggressive antihypertensive therapy and risk for adverse clinical outcomes.

To avoid such bias caused by potential confounding factors, observational studies routinely adjust for baseline confounding variables. However, in the case of hypertension and its step-up therapy protocols, the problem is complicated as both treatment (antihypertensive drugs) and confounder (blood pressure) can change over time (are time-dependent) and the confounder acts as an important mediating variable through which the treatment exerts its beneficial effect. In the presence of this type of time-dependent confounding, standard methods of confounder adjustment do not produce unbiased estimates [7].

**Figure 1:**
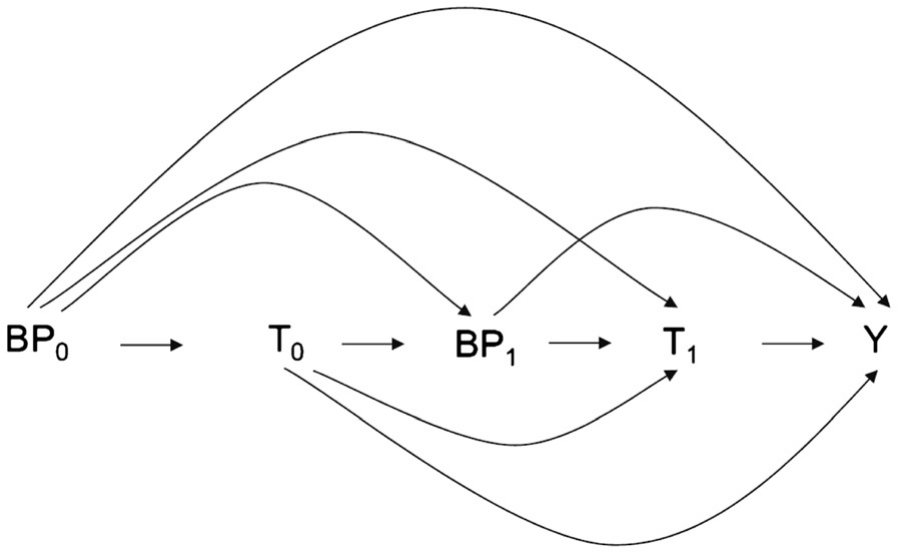
**Directed acyclic graph illustrating time-dependent confounding by blood pressure in the treatment of hypertension.** Abbreviations: →, causal effect; BP0, blood pressure at time 0; BP1, blood pressure at time 1; T0, antihypertensive treatment at time 0; T1, antihypertensive treatment at time 1; Y, cardiovascular event.

Figure 1 illustrates the theoretical foundation of the type of time-dependent confounding described above. Let blood pressure (BP) be a time-dependent confounder, T be the time-dependent treatment (e.g., antihypertensive drug treatment of varying intensity) and Y be the outcome (e.g., a cardiovascular event), with subscripts 0 and 1 denoting two time points during follow-up. If blood pressure, the time dependent confounder, is not controlled for in the analysis, then blood pressure at time 1 confounds the association of treatment at time 1 with the outcome, because it simultaneously affects both treatment at time 1 and the risk for a cardiovascular event. Thus, any estimate of the association of antihypertensive treatment with the risk for cardiovascular events would be biased if blood pressure is not controlled for. However, if blood pressure is controlled in the analysis through conventional statistical methods, then blood pressure at time 1, a variable in the causal path of the effect of antihypertensive treatment at time 0 on cardiovascular event risk, is adjusted for, again, resulting in a biased estimate of the causal effect of antihypertensive treatment on cardiovascular event risk. While conventional statistical models fail to produce unbiased estimates under these conditions, marginal structural models (MSMs), first introduced by Robins et al., are designed to produce unbiased estimates in the presence of time-dependent confounding [7].

Previous studies have applied MSMs to similar problems in other clinical areas, including arthritis, cardiovascular disease, and HIV [8-10]. The utility of MSMs has also been examined in the context of hypertension, [11,12] providing empirical support for the claim that a MSM approach is appropriate for observational studies of antihypertensive medication use.

This study aims to estimate the effect of aggressive (≥3 concomitant antihypertensive drugs) vs. conventional (≤2 concomitant antihypertensive drugs) antihypertensive therapy on serious adverse cardiovascular outcomes in the 22,576 patients enrolled in the INternational VErapamil-Trandolapril STudy (INVEST) using a Marginal Structural Cox Model (MSCM) to account for time-dependent confounding by systolic blood pressure and differential loss to follow-up over the course of the study. By comparing the results of the MSCM to the results of a standard Cox model, we aim to determine the extent of bias that might be introduced by the use of standard methods.

## Methods

### Study population

The present investigation is a post-hoc analysis of data from INVEST, a large, international, randomized controlled hypertension treatment trial that enrolled patients with hypertension and coronary artery disease (CAD) between January 1998 and February 2001. Details of the design, rationale and results have been previously published [13,14]. Briefly, after an extensive cardiovascular history and physical exam, 22,576 CAD patients ≥50 years old were randomly assigned to either a verapamil SR- or an atenolol-based multidrug antihypertensive strategy. Trandolapril and hydrochlorothiazide (HCTZ) were specified as added agents, if needed for blood pressure control, with trandolapril added first in the verapamil SR strategy and HCTZ added first in the atenolol strategy. In both strategies, trandolapril was recommended for organ protection among patients with heart failure, diabetes, or renal impairment. Follow-up continued until a patient was lost to follow-up, died, or the end of the study. A total of 61,835 patient-years were accumulated and both strategies provided excellent blood pressure control (>70% of patients achieved blood pressure <140/90 mm Hg) with no difference in mean blood pressure between treatment strategies at any of the follow-up visit time points. Additionally, there was no significant difference between the strategies in preventing the primary outcome (hazard ratio 0.98, 95% confidence interval 0.90-1.06), which was the first occurrence of all-cause death, nonfatal myocardial infarction (MI), or nonfatal stroke. For the present analyses, patients from both treatment strategies were combined. The protocol was conducted in accordance with principles outlined in the Declaration of Helsinki, and institutional review boards and ethics committees at participating sites approved the protocol. All patients provided written informed consent.

### Study variables

During follow-up, the protocol directed scheduled visits every six weeks for the first six months and every six months until the last patient enrolled was followed for two years. To decrease visit burden, patients with controlled blood pressure at visits 2 and 3 were allowed to skip visit 4 and then be seen at visit 5. The largest possible number of visits included in this analysis was 14. At each visit, patients were assessed for occurrence of adverse events, and response to treatment. If patients did not return for one or multiple protocol visits, values for study variables (e.g., systolic blood pressure, antihypertensive drug use) were carried forward from the last available visit. If a patient was lost to follow-up (i.e., did not have another visit or final assessment), the patient was censored at the time of the last visit.

### Exposure

Antihypertensive drug use was recorded at each visit for all study- (atenolol, verapamil, HCTZ, trandolapril) and non-study antihypertensive drugs (all other antihypertensive drugs). For this study, both study and non-study antihypertensive drugs were included in the analysis. At each visit, we defined aggressive and conventional treatment as simultaneous exposure to ≥3 and ≤2 antihypertensive medications, respectively. This definition was chosen empirically based on the average number of antihypertensive drugs used in INVEST which, depending on study visit, ranged between two and three [14]. To assess robustness of our results in regards to this exposure definition, two sensitivity analyses were conducted that defined aggressive treatment as ≥4 (vs. ≤3) and ≥2 (vs. ≤1) concurrently used antihypertensive drugs.

### Outcome

The primary outcome for the present analysis is the same as that used by the original INVEST protocol, and was defined as the first occurrence of all-cause death, nonfatal MI, or nonfatal stroke. All components of this composite outcome were fully adjudicated by an independent end point committee.

### Covariates

Baseline covariates included in the analysis include age, sex, race/ethnicity, randomized treatment strategy, and baseline history of myocardial infarction, stroke/transient ischemic attack, congestive heart failure, diabetes, renal impairment, peripheral vascular disease, CABG/PCI, or smoking [15]. Blood pressure was measured twice, at least 2 minutes apart, at each visit with a standard mercury sphygmomanometer in a sitting position. [16] In a given patient throughout the trial all measurements were taken on the same arm, and, when possible, approximately the same time of day to minimize diurnal variation and measurement error. Only systolic blood pressure was included in this analysis. Because of the J-shaped relationship between systolic blood pressure and the risk for cardiovascular outcomes in INVEST, [17-19] systolic blood pressure was categorized into three categories, tight control (<120 mm Hg), usual control (120 mm Hg to <140 mm Hg), and not controlled (≥ 140 mm Hg).

### Statistical methods

The effect of aggressive antihypertensive treatment on the risk of the primary outcome was assessed adjusted for time-dependent systolic blood pressure using a Cox proportional hazards regression with combined stabilized weights (i.e., a marginal structural Cox model), as well as unadjusted for time-dependent systolic blood pressure using a Cox proportional hazards regression without weights (i.e., a standard time-dependent Cox model).

### Marginal structural models

MSMs use inverse probability of treatment weights (IPTWs) and inverse probability of censoring weights (IPCWs) to create a pseudo-population in which treatment is un-confounded by subject specific characteristics and no censoring occurs [10]. MSMs are fitted in a two-stage process. The first step estimates the individual IPTWs and IPCWs. The IPTWs are based on each subject’s probability of having their own treatment history at each time point given the subject’s covariates (including time-dependent covariates). The IPCWs are similarly estimated based on each subject’s probability at each time point to be censored based on his or her covariates. The second step uses the IPTWs and IPCWs as weights in a regression model of the effect of the treatment on the outcome. Because of the weighting, the regression now takes place in the pseudo-population and results — under the assumption of no unmeasured confounding and correct model specifications — in a causal estimate of the treatment’s effect on the study outcome.

For this study, a MSCM was used to estimate the association between time-dependent antihypertensive treatment (aggressive vs. conventional treatment) on the primary outcome, controlling for systolic blood pressure at each visit. First, stabilized IPTWs and IPCWs were estimated, as stabilized weights have been shown to produce narrower confidence intervals with better coverage rates than unstablized weights [7,10]. The IPTWs were estimated using a pooled logistic regression model for the probability of being exposed to aggressive versus standard antihypertensive therapy at visit (k) conditional on select baseline covariates and systolic blood pressure (tight control, usual control, not controlled) at baseline, visit (k) and visit (k-1). The IPCWs were estimated in the same fashion using a pooled logistic regression model for the probability of being censored at visit (k). Lastly, combined stabilized weights were computed by multiplying the IPTW and IPCW for each patient visit and used in a weighted Cox proportional hazards model. To overcome computational limitations of standard software which cannot typically handle time-varying weights in Cox proportional hazards models, the hazard ratio was estimated by fitting a pooled logistic regression model that included the weights, baseline covariates and the time-dependent antihypertensive treatment variable. [10,20] SAS 9.1 (SAS Institute, Carey, NC) was used for data management and analysis. SAS code provided by Hernan et al. was adapted and used to fit the MSCMs for our study [10]. This approach required a binary independent variable, assumed that, once started, aggressive treatment was continued until censoring, and assumed that observations were equally spaced.

### Time-dependent Cox proportional hazards models

We also estimated the hazard ratio for aggressive vs. conventional antihypertensive treatment with a standard time-dependent Cox model using the same pooled logistic regression model that was used in the MSCM above but without inverse probability weighting. The hazard ratio obtained by the conventional time-dependent Cox model was then compared to the one obtained from the MSCM to determine the magnitude of the potential confounding effect of systolic blood pressure on the hazard ratio for aggressive vs. conventional antihypertensive treatment.

## Results

The 22,576 patients enrolled in INVEST accrued a total of 61,845 patient years of follow up, with 2,269 patients experiencing a primary outcome event during this period. For the present investigation, 906 patient years of follow-up were excluded because they accrued after the occurrence of a nonfatal primary outcome event and thus, a total of 60,939 patient years of follow-up remained available for analysis. Baseline characteristics are summarized in Table 1.

**Table 1:** Pertinent Characteristics of INVEST Patients at Baseline

**Variable**	**N = 22576**
Demographic	
Age, mean (SD), years	66.1 (9.8)
Women	11770 (52.1)
Race/Ethnicity	
White	10925 (48.4)
Black	3029 (13.4)
Hispanic	8045 (35.6)
Other/multiracial	577 (2.6)
Verapamil SR Strategy	11267 (49.9)
Atenolol Strategy	11309 (50.1)
History of	
Myocardial infarction	7218 (32.0)
Stroke/transient ischemic attack	1629 (7.2)
Congestive Heart Failure (class I-III)	1256 (5.6)
Diabetes†	6400 (28.4)
Renal impairment‡	424 (1.9)
Peripheral vascular disease	2699 (12.0)
CABG or PCI	6166 (27.3)
Smoking (ever)	10454 (46.3)
Blood Pressure Systolic Blood Pressure, mean (SD)	149.5 (19.7)
Diastolic Blood Pressure, mean (SD)	86.3 (12.0)
Heart Rate, mean (SD), beats/min	75.5 (9.5)

Abbreviations: SD, Standard deviation; CABG, Coronary artery bypass graft; PCI, percutaneous coronary interventions.

Unless indicated otherwise, values express numbers (percentage).

†History of or currently taking antidiabetic medications.

‡History of or currently have elevated serum creatinine level but less than 4 mg/dl (354 μmol/L).

Table 2 provides an inverse probability of treatment and censoring weighted estimate for the effect of aggressive (≥3 concurrent antihypertensive drugs) versus conventional (≤2 concurrent antihypertensive drugs) antihypertensive treatment, compared with estimates from the equivalent standard time-dependent Cox model. The standard Cox model estimated no statistically significant difference in the primary outcome comparing treatment with aggressive vs. conventional antihypertensive treatment (HR 0.96, 95% CI 0.87-1.07). In contrast, the MSCM estimated a statistically significant 19% (HR 0.81, 95% CI 0.71-0.92) reduction in the hazard for the primary outcome for patients treated with aggressive antihypertensive therapy as compared with conventional therapy.

**Table 2:** Effect of aggressive antihypertensive treatment on INVEST primary outcome: Estimates from a marginal structural Cox model and a standard Cox model

	***Hazard Ratio***	***95% Confidence Interval***
**Primary Analysis**		
Conventional treatment (≤ 2 antihypertensive drugs)	1.0	
Aggressive treatment (≥3 antihypertensive drugs)		
Marginal structural Cox model	0.81	0.71-0.92
Standard Cox model	0.96	0.87-1.07
**Sensitivity Analysis 1**		
Conventional treatment (≤ 1 antihypertensive drugs)	1.0	
Aggressive treatment (≥2 antihypertensive drugs)		
Marginal structural Cox model	0.83	0.64-1.06
Standard Cox model	0.89	0.70-1.13
**Sensitivity Analysis 2**		
Conventional treatment (≤3 antihypertensive drugs)	1.0	
Aggressive treatment (≥4 antihypertensive drugs)		
Marginal structural Cox model	0.79	0.71-0.89
Standard Cox model	0.98	0.90-1.07

Table 2 also shows the results of two sensitivity analyses conducted to assess whether our results are stable across additional exposure definitions. Across all exposure definitions, the MSCM consistently estimated a larger beneficial effect for aggressive treatment than the standard Cox model. The between-model differences (MSCM vs. standard Cox model) in HR estimates grew in magnitude (from 0.06 to 0.19) as the number of concomitant drugs required for the aggressive treatment definition increased from ≥2 to ≥4.

## Discussion

Our findings suggest that in hypertensive patients with CAD, aggressive antihypertensive therapy is associated with reduced risk for adverse cardiovascular outcomes compared with more conventional treatment. This effect was only apparent when using a MSCM that adjusted for time-dependent confounding by systolic blood pressure and differential loss to follow-up, not when a standard Cox model was used. This suggests that time-dependent confounding by systolic blood pressure and/or differential loss to follow-up introduces bias into the estimates of treatment effects for antihypertensive combination therapies resulting in an under-estimation of the benefits of more aggressive treatment regimens. Similar findings were observed across the two additional exposure specifications used in sensitivity analysis. However, the observed differences between the MSCMs and standard Cox models did not rise to statistical significance in two out of the three exposure specifications. The magnitude of the between model differences increased with the number of concomitant drugs required for the aggressive treatment definition, suggesting that time-dependent confounding by blood pressure may be of increasing concern the more aggressive the examined treatment strategy becomes.

Assuming a correctly specified model and no violation of the underlying model assumptions, the MSCM estimate for the effect of aggressive versus conventional antihypertensive therapy is interpretable as the effect that would have been observed in a RCT that compared the effects of aggressive to conventional treatment without making any adjustments in therapy to assure blood pressure control. In the absence of direct experimental evidence on treatment intensity, our results thus suggest that more aggressive treatment strategies are beneficial in adults older than 50 years with hypertension and prevalent CAD. Importantly, this finding is limited in its clinical utility as antihypertensive therapy in practice, as well as in typical RCTs, is highly dynamic (i.e., routinely adjusted to achieve and maintain target blood pressure goals) while our analysis makes inferences about a static treatment comparison. Our findings also need to be applied in the context of the known fact that more complex treatment regimens generally result in lower adherence [21] and increased risk of adverse effects [22].

Our results are consistent with other studies that have considered blood pressure as a mediating, time-dependent confounder [11,12]. Specifically, our results extend the findings by *Sugihara* et al*.* from blood pressure response to clinical outcomes, and suggest that aggressive management of hypertension may not only result in higher achievement rates of target blood pressure [12] but also in clinically important reduction in cardiovascular outcomes. In these previous studies, the use of IPTW to handle time dependent confounding by blood pressure enabled observational analyses of key associations that were consistent with the associations found from RCTs. In contrast, conventional approaches led to significant amounts of bias in the estimation of the association between antihypertensive medications and cardiovascular outcomes [11,12]. The previous work on this subject as well as the known theoretical results give us confidence that a MSM approach is providing the best estimate of the effectiveness of aggressive antihypertensive therapy and is less susceptible to bias when compared with standard analytical approaches.

Of course, there are other possible explanations for the observed differences between the two approaches. The estimates from a MSCM are marginal estimates and thus estimate the contrast between treating everyone and treating no-one. In contrast, the conventional estimates are conditional estimates and thus estimate the effect of treatment holding all other variables constant. In the presence of non-linear covariates or effect measure modification, these estimators will be estimating different parameters which could explain some of the observed differences. The MSCM approach also accounted for loss to follow-up (via IPCW) whereas the conventional approach assumed random censoring.

Importantly, our results do not speak to the mechanisms by which aggressive antihypertensive therapy reduces the risk for cardiovascular outcomes, which may include both non-blood pressure mediated effects of individual agents, e.g., heart rate lowering effects of beta blockers in patients with a history of myocardial infarction [23], as well as tighter blood pressure control. That tight blood pressure control might be of benefit to patients with hypertension and CAD is not unexpected, as strong evidence suggests that the cardio-protective effects of blood pressure lowering medications are largely mediated by blood pressure reduction [24-27].

This study illustrates how properly constructed and appropriately analyzed observational studies can provide some clinical guidance until direct RCT evidence is available. Further extensions of this work could aim to increase clinical utility by using dynamic treatment regimens that consider specific blood pressure thresholds for aggressive versus conventional treatment to allow identification of high-risk sub-groups [28,29], as there has been some evidence that aggressive treatment may be less effective in some sub-populations [18], and examine specific drug and drug/dose combinations.

### Limitations

The present study has several limitations. First, the generalizability of our study is limited to hypertensive patients with CAD. However, this population represents a large and rapidly growing important high risk population that has not been at the center of antihypertensive research efforts and therefore warrants comprehensive investigation [30].

Second, measurement error is inherently problematic in studies of hypertension [31-33]. While the INVEST protocol tried to minimize measurement error by providing to all site investigators standardized blood pressure measurement instructions following JNC VI guidelines [34], some measurement error is unavoidable. However, it is unlikely that this measurement error results in important levels of bias and more likely reduces the precision of the estimates due to additional random error in the estimates.

Third, we imputed data for missing visits during follow-up which may result in limited misclassification of both the exposure and the time-dependent confounder. Such misclassification would likely bias results (effect estimates of aggressive vs. conventional therapy as well as differences between the two analytic approaches) towards the null.

Fourth, to simplify the analytic approach, we focused the adjustment for time-dependent confounding on systolic blood pressure, and did not include additional time-dependent variables such as diastolic blood pressure or heart rate. More complex modeling of multiple time-dependent confounding factors is an active area for future research in this field.

The use of a MSCM to adjust for time-dependent confounding through inverse probability of treatment and censoring weighting introduces additional limitations to the study. MSMs, as implemented in this study, require a number of assumptions and a rather simple data structure. First, the independent variable of interest has to be binary. Our study categorized antihypertensive drug use into aggressive vs. conventional antihypertensive treatment. This definition does not distinguish between specific drugs and/or drug classes nor does it take dosing of each specific drug into account. If treatment effects vary between specific drugs and doses, our model will not show these differences and rather produce an estimate reflective of the average treatment combinations used in the aggressive and conventional treatment groups. Thus, our results do not address the question of the optimal selection or dosing of specific antihypertensive drugs for the aggressive therapy regimen.

Second the method requires the assumption, that once initiated, exposure is not discontinued until the end of follow-up or censoring (an intention to treat approach to exposure). This assumption is not fully met in INVEST using actual medication use. We therefore focused on the decision to begin aggressive (as opposed to conventional) treatment as our object of inference rather than an “as treated” analysis, which likely makes the analysis more conservative (i.e., introduces a bias towards the null).

Third, the pooled logistic regression model that was used to estimate the MSCM assumes that observations are equally spaced, which was not the case in the INVEST where the initial five visits occurred in six week intervals while the remaining visits occurred in six month intervals.

Lastly, as discussed in more detail in the introduction, in the presence of a time-dependent confounder standard methods allow two options, both expected to result in biased estimates — to disregard the time-dependent confounder (and accept the resulting bias introduced by the time-dependent confounder) or to adjust for the time-dependent confounder (and introduce bias by adjusting for a variable on the causal pathway) [7]. Our study focused on the comparison between the MSCM to the former standard option, i.e., compared the MSCM to a standard Cox model that allowed exposure to vary over time but held all covariates, including systolic blood pressure, fixed at their baseline value. We chose this comparison because the standard Cox model with fixed covariates is more widely used and because its comparison to the MSCM provides a direct estimate of the bias introduced by the time-dependent confounder which is more readily interpretable than the bias introduced by inappropriate adjustment (adjustment for a time-dependent intermediate variable that is affected by prior treatment). However, the alternative approach (a comparison to a standard Cox model that allows both exposure and covariates to change over time) [35] could be considered fairer as the both approaches ‘adjust’ for the time-dependent confounder and may, in our example, have shown better performance in terms of bias. One should therefore be cautious in generalizing the estimates of bias provided in this paper to scenarios involving Cox models with time-dependent covariates.

### Summary and conclusions

It is conceptually clear that differential loss to follow-up and time-dependent confounding by a surrogate are problematic for the estimation of treatment effects in observational studies. Our findings empirically illustrate this issue by showing that standard Cox models systematically underestimate the effectiveness of aggressive antihypertensive combination treatment in a post-hoc analysis of a large hypertension treatment RCT and thus demonstrate the importance of using appropriate methods to address time-dependent confounding and differential loss to follow-up in observational antihypertensive drug studies. Although they do not support inferences regarding specific mechanisms of action, the results from a MSCM clearly suggest that more aggressive antihypertensive regimens are associated with a reduced rate of serious cardiovascular events in patients with hypertension and prevalent CAD.

## Competing interests

Drs. Pepine and Cooper-DeHoff received research grants and Dr. Pepine consulted for Abbott Laboratories. The other authors have no financial interests to disclose. Abbott Laboratories had no role in the design or conduct of the study, collection or analysis of the data, or preparation or approval of the manuscript.

## Authors’ contributions

TG conceived of the study, led its design, performed the statistical analysis, and wrote the draft manuscript. JD contributed to the data interpretation and drafting of the manuscript, and reviewed the draft manuscript for intellectual content. RC facilitated the access to the INVEST data and reviewed the draft manuscript for intellectual content. JS participated in the design of the study, oversaw the statistical analysis, and reviewed the draft manuscript for intellectual content. BB participated in the design of the study, oversaw the statistical analysis, contributed to the data interpretation and reviewed the draft manuscript for intellectual content. JJ participated in the design of the study and reviewed the draft manuscript for intellectual content. CP facilitated the access to the INVEST data, participated in the design of the study, and reviewed the draft manuscript for intellectual content. AW oversaw the design and all aspects of the study, and reviewed the draft manuscript for intellectual content. All authors read and approved of the final manuscript.

## Pre-publication history

The pre-publication history for this paper can be accessed here:

http://www.biomedcentral.com/1471-2288/12/119/prepub
